# Supplementary material for: WordCommentsAnalyzer: A windows software tool for qualitative research
Source: F1000Res. 2018 Sep 4;7:536. Originally published 2018 May 3. [Version 2] doi: 10.12688/f1000research.14819.2 (PMC6137411; doi:10.12688/f1000research.14819.2)
Supplement: Supplementary file 3 [file f1000research-7-17614-s0002.tgz › b2a79496-4ada-47c4-9682-a99ce867836d.zip!/Eight Iranian Health Journals/Journal of Knowledge and Health_14.docx]

© Journal of Knowledge and Health. This work is licensed under CC BY 4.0 (https://creativecommons.org/licenses/by/4.0/).

Volume & Issue: دوره 9، شماره 4: 1393

Title: تأثیر ماساژ پا بر میزان ادم فیزیولوژیک بارداری

http://knh.shmu.ac.ir/index.php/site/article/view/493

مقدمه: یکی از مشکلات شایع و آزاردهنده در دوران بارداری ادم فیزیولوژیک پا مي‌باشد که باعث ایجاد محدودیت‌هایی در فعالیت زن باردار مي‌گردد. پژوهش حاضر به‌منظور تعیین تأثیر ماساژ بر میزان ادم فیزیولوژیک بارداری انجام شده است. مواد و روش‌ها: این پژوهش از نوع مطالعات کارآزمایی بالینی شده بود که در سال 1391 انجام شد. در این مطالعه، 120 زن باردار در محدوده سنی 20 تا 35 سال به‌طور غیرتصادفی در دو گروه آزمون و کنترل قرار گرفتند. افراد گروه آزمون، 20 دقیقه ماساژ روزانه پاها در طی 5 روز دریافت نمودند. داده‌ها با استفاده از آزمون‌های تی مستقل و من ویتنی یو با نرم‌افزار آماری SPSS، مورد تجزیه و تحلیل قرار گرفت. پیامد این مطالعه، سنجش میزان ادم پا، توسط متر نواری غیرقابل ارتجاع بود. نتايج: نتايج اين پژوهش اختلاف آماری معناداري را بین میانگین میزان محیط‌های اندازه‌گيري شده در پاها (دور مچ، پاشنه و مفصل میان انگشتان و استخوان‌های متاتارس) در دو گروه آزمون و کنترل نشان داد (001/0P<). نتیجه‌گیری: نتایج این مطالعه نشان داد که ماساژ درمانی در کاهش ادم فیزیولوژیک بارداری مؤثر است. بنابراين ماساژ اندام تحتانی مي‌تواند به‌عنوان یک روش مفید، کم خطر و کم هزینه توسط ماماهای آموزش دیده در مراكز ارائه‌دهنده مراقبت‌های قبل از زایمان و یا در منازل زنان باردار انجام شود.

Title: مدل اختلال حافظه در بیماری پارکینسون با تخریب دوطرفه بخش متراکم جسم سیاه در موش صحرایی

http://knh.shmu.ac.ir/index.php/site/article/view/411

مقدمه: تا مدت‌ها پیش تنها اختلالات حرکتی بیماری پارکینسون مورد توجه محققین بود، ليكن اختلالات شناختی و حافظه از علائم غیر‌حرکتی این بیماری است که قبل از علائم کلینیکال ظاهر مي‌گردند. لذا در این تحقیق تزریق نوروتوکسین 6-هیدروکسی دوپامین (6-OHDA) در مناطق متراکم جسم سیاه دو طرف مغز انجام شدكه با‌توجه به ارتباط عصبی بین جسم سیاه مغز میانی و هیپوکمپ، منجر به استرس اکسیداتیو در هیپوکمپ گردیده متعاقب آن مدل اختلال حافظه پارکینسون ایجاد گرديد. مواد و روش‌ها: 6 میکروگرم نوروتوکسین6-OHDA  به بخش متراکم جسم سیاه به‌صورت دوطرفه تزریق شد (گروه آسیب). به گروه شم نیز نرمال سالین به جای نوروتوکسین تزریق شد. آزمون فراگیری در روزهای 9 تا 12 پس از جراحی، با آزمون ماز آبی موریس ارزیابی شد و سپس 24 ساعت بعد از آخرین آموزش، آزمون پروب گرفته شد. سرانجام حیوانات قربانی شده و پس از جدا سازی هیپوکمپ، فعالیت آنزیم‌های سوپراکسید دیسموتاز SOD))، گلوتاتیون پراکسیداز(GPX)  و کاتالاز CAT)) مورد بررسی قرار گرفت. نتايج: اختلاف معني‌داری در کل مسافت پیموده شده بین دو گروه مشاهده نشد، که نشان‌دهنده‌ی عدم‌وجود اختلالات حرکتی در گروه آسیب بود. در روز پروب افزایش معني‌داری در زمان رسیدن به محل سکو در گروه آسیب نسبت‌به گروه شم مشاهده شد (001/0P<). درصد زمان حضور در ربع هدف در گروه آسیب نسبت‌به گروه شم کاهش معني‌داری پیدا کرده بود (002/0>P). آنالیز واریانس یک طرفه، کاهش معني‌دار فعالیت آنزیم SOD در گروه آسیب نسبت‌به گروه شم را نشان داد (05/0P<)، ولی این کاهش در رابطه با آنزیم‌های  GPXو  CATمعني‌دار نبود. نتيجه‌گيري: تخریب دوطرفه بخش متراکم جسم سیاه به‌عنوان يك مدل حيواني در مراحل اوليه‌ي بيماري پاركينسون مي‌تواند براي بررسي اثر نوروپروتکتیو بسیاری از داروهای آنتی‌اکسیدان و روش‌های درمانی مناسب مورد آزمون قرار گيرد.

Title: اثرات استات سرب طی دوره تکوینی پس از تولد بر مخچه موش‌های صحرایی نر: ارزيابي بافت‌شناسي و رفتاری

http://knh.shmu.ac.ir/index.php/site/article/view/338

مقدمه: مسمومیت حاد یا مزمن حیوانات و انسان‌ها با سرب حتي با غلظت پايين به‌خوبی شناخته شده است. همچنين بررسي‌هاي اخير نشان داد كه مغز در حال تکوین نيز آسيب‌پذيری ویژه‌اي نسبت‌به مسمومیت عصبی با سرب دارد. از‌آنجا‌که تکوین مخچه تا مدتي پس از تولد ادامه مي‌يابد، اين مطالعه با هدف تأثير سرب بر ساختار بافتی قشر مخچه و رفتارهای حرکتی موش‌های صحرایی طی دوره تکوینی پس از تولد طراحي گرديد. مواد و روش‌ها: در این مطالعه تجربی نوزادان نر موش‌های صحرایی به تصادف به دو گروه کنترل (10=n) و در معرض سرب (10=n) تقسیم شدند. گروه در معرض سرب، استات سرب را با دوز mg/kg 8 از روز اول تا 21 روز پس از تولد روزانه یک‌بار به‌صورت تزریق داخل صفاقی دریافت کردند. مطالعات رفتاری شامل آزمون‌های روتارود و open field در روزهای 33-31 پس از تولد و مطالعات بافتی بعد از پایان آزمون‌های رفتاری در روز 33 انجام گرفت. نتايج: تيمار با سرب موجب اختلال در اجرای آزمون روتارود در جلسات 1، 2 (01/0>P) و 4، 6 (05/0>P) گرديد. همچنین سرب باعث اختلال در فعالیت‌های حرکتی و جستجوگرانه حیوانات گردیده به‌طوری‌که تعداد مربع‌های پیموده شده (05/0>P) و تعداد ایستادن‌ها (05/0>P) در آزمون Open field به‌طور معني‌داري كاهش يافت. نتایج حاصل از مطالعات بافتی نيز نشان داد که تعداد سلول‌های پورکنژ به میزان قابل‌توجهی در گروه تیمار شده با سرب نسبت‌به کنترل کاهش مي‌يابد (001/0=P). نتیجه‌گیری: این نتایج پیشنهاد مي‌کند که قرارگيري در معرض سرب طی دوره تکوینی پس از تولد، آثار سوء بر مخچه داشته و باعث اختلال در اجراي فعالیت‌های حرکتی و رفتار جستجوگرايانه موش‌هاي صحرايي مي‌گردد.

Title: ارزشیابی تحقق اهداف آموزش مهارت‌های بالینی پایه پرستاری به‌روش آسکی در دانشجویان سال آخر پرستاری در بخش ICU

http://knh.shmu.ac.ir/index.php/site/article/view/370

مقدمه: آموزش باليني ركن اساسی و قلب آموزش پرستاري محسوب مي‌گردد. پرستاری حرفه‌ای مبتنی بر عملکرد می‌باشد و ارزشیابی تحقق اهداف آموزشی دانشجویان پرستاری در شکل کسب مهارت از ضروریات این حرفه می‌باشد. هدف این تحقیق تعیین میزان دستیابی به اهداف آموزشی بالینی در مهارت‌های بنیادین پرستاری در دانشجویان سال آخر پرستاری در بخش مراقبت‌های ویژه بود. مواد و روش‌ها: این پژوهش به‌صورت توصیفی- تحلیلی بر روی 56 نفر از دانشجویان پرستاری سال آخر صورت گرفت. نمونه‌ها به‌صورت هدفمند در بخش مراقبت‌های ویژه انتخاب شدند و در ایستگاه‌های آسکی تحت مشاهده مستقیم قرار گرفتند. نتایج به‌دست آمده از برگه مشاهده با استقاده از نرم‌افزار SPSS نسخه 19مورد تجزیه و تحلیل قرار گرفت. در هر مهارت، سطح توانايي دانشجويان در پنج سطح از غیرقابل قبول تا عالي رده‌بندي شد. نتايج: در بررسي نتايج به‌دست آمده از اين پژوهش مشخص مي‌گردد كه تنها در رويه پرستاري گذاردن آنژيوكت دانشجويان پرستاري سطح مهارت بسيار خوب يا عالي را نشان دادند در‌حالي‌كه هيچ يك از واحدهاي مورد پژوهش در برخي مهارت‌ها از قبيل پانسمان، گذاشتن لوله معده و سوند ادراري سطح مهارت بسيار خوب يا عالي را نشان ندادند. تنها متغیری که با مهارت دانشجویان رابطه معنی‌داری داشت میزان فرصت‌های تکرار و تمرین رویه پرستاری در آموزش باليني بود (01/0=P). نتيجه‌گيري: در اين پژوهش مشخص گرديد كه دانشجويان پرستاري در انجام مهارت‌هاي پايه باليني دچار نقايص قابل توجهي بودند. با‌توجه به اهمیت پرورش مهارت‌هاي پايه باليني دانشجويان پرستاري، پيشنهاد مي‌گردد فرصت‌های یادگیری بیشتری برای دانشجویان فراهم گردد تا به تمرین مهارت‌های پایه بالینی در دوران آموزشي بپردازند.

Title: تحریک تخمک‌گذاری با FSH ادراری و نوترکیب: مقایسه میزان بارداری در بیماران نابارور مبتلابه سندرم تخمدان پلی‌کیستیک

http://knh.shmu.ac.ir/index.php/site/article/view/394

مقدمه: یکی‌‌‌از علل ناباروری، عدم تخمک‌گذاری به‌دلیل سندرم تخمدان پلی‌کیستیک (PCOS) می‌‌باشد. در زنان مبتلا‌به PCOS، عموماً غلظت بالای سرمی LH و افزایش نسبت LH به FSH دیده می‌شود. به‌منظور پيشگيري از افزايش LH و آترزي فوليكولي، استفاده از FSH پيشنهاد گرديده است. هدف از مطالعه‌ حاضر، مقایسه میزان بارداري در كاربرد دو رژيم درماني  FSH نوتركيب (r-FSH) و FSH ادراري (u-FSH) در سیکل‌های IUI در بیماران مبتلابه PCOS می‌باشد. مواد و روش‌ها: این مطالعه به‌صورت توصیفی- تحلیلی (گذشته‌نگر) برروي 441 سیکل بیمار نابارور مبتلابه PCOS مراجعه‌کننده به کلینیک نازایی بیمارستان شهید بهشتی کاشان (طي دو سال) واجد شرايط ورود به مطالعه، با مراجعه به پرونده‌های آنان انجام گرفته است. نتايج: از نظر میانگین ضخامت آندومتر، تعداد فولیکول غالب، میزان گنادوتروپین مصرفی، مدت و نوع نازايي اختلاف آماری معني‌داری بين دو گروه وجود نداشت. میزان بارداری در بیماران تحت درمان با u-FSH، 6/28 درصد و در بیماران تحت درمان با r-FSH، 9/30 درصد بود که این اختلاف از نظر آماری معني‌دار نبود (59/0P=). نتيجه‌گيري: يافته‌هاي پژوهش حاضر، نشان داد که بین دو فرآورده u-FSH و r-FSH در بیماران نابارور مبتلابه PCOS در سیکل‌های IUI، از نظر میزان باروری تفاوتی وجود ندارد. از اين رو، مي‌توان از هر دو این فرآورده با‌توجه به در دسترس بودن، پذیرش بیمار و هزینه مصرفی استفاده نمود.

Title: مقايسه استحصال و موفقيت در تخليص سلول‌هاي بنيادي مزانشيمي از دو منبع مغز استخوان و بافت چربي انسان

http://knh.shmu.ac.ir/index.php/site/article/view/320

مقدمه: سلول‌هاي بنيادي مزانشيمي (MSCs: Mesenchymal stem cells) با داشتن مزيت‌هايي نظير قابليت توان تمايزي و اعمال آثار پاراكرين در درمان بیماری‌های مختلف نقش دارند. لذا استفاده از MSCs به‌عنوان ابزاري در سلول درماني، نیازمند استحصال موفق این سلول‌ها در كمترين زمان ممكن است. مواد و روش‌ها: به همین منظور در اين مطالعه MSCs از منابع مغز استخوان و چربی انسان استخراج شده و از نظر درصد موفقيت كشت و سرعت تخليص مورد ارزیابی قرار گرفتند. برای اطمینان از اینکه سلول‌های استحصال شده MSCs هستند، مطالعه تمایز به سلول‌های چربی و سلول‌های استخوانی به‌وسيله رنگ‌آميزی Oil Red O و Alizarin Red S و سنجش فعالیت آلکالین فسفاتازی انجام گرفت. همچنین سلول‌های جدا شده، از نظر نشانگرهای اختصاصی به‌وسيله فلوسایتومتری مورد بررسی قرار گرفتند. نتايج: نتایج حاكي از آن بود که درصد موفقيت در جداسازي MSCs از بافت چربي بيشتر از بافت مغز استخوان بود و همچنين روند تخليص در MSCs جدا شده از بافت چربي در مقايسه با MSCs جدا شده از مغز استخوان به‌مدت زمان كمتري نياز دارد. همچنين نتایج ما مويد اين واقعيت بود که استحصال MSCs از بافت چربی از دهنده‌های مختلف كاملاً تکرارپذیر بوده و وابسته به دهنده نيست ليكن اين مسأله در مورد MSCs مشتق از بافت مغز استخوان صادق نبود.نتيجه‌گيري: در مجموع نتايج اين تحقيق بيانگر اين بود كه بافت چربي در مقايسه با بافت مغز استخوان، به لحاظ درصد موفقيت در جداسازي اوليه سلول‌ها و همچنين سرعت تخليص بعدي سلول‌ها، منبع مناسب‌تري برای استخراج MSCs مي‌باشد.

Title: پیش‌بینی نشانگان بالینی در دانشجویان دارای تجربه شکست عاطفی بر اساس ساختارهای شخصیتی: نقش تعدیل‌کننده‌ی حمایت اجتماعی ادراک شده

http://knh.shmu.ac.ir/index.php/site/article/view/397

مقدمه: هدف پژوهش حاضر، بررسی نقش ساختارهای شخصیتی در پيش‌بيني نشانگان بالینی در دانشجویان دارای تجربه شکست عاطفی و نقش تعدیل‌کنندگی حمایت اجتماعی ادراک شده در این رابطه مي‌باشد. مواد و روش‌ها: با استفاده از روش نمونه‌گیری هدفمند و براساس نمرات افراد دارای تجربه شکست عاطفی در پرسش‌نامه‌های ضربه عشق، افسردگی بک و سلامت روان، 65 دانشجوی دارای نشانگان بالینی و 65 دانشجوی بدون نشانگان بالینی از دانشگاه‌های پیام نور تبریز، دانشگاه تبریز، دانشگاه آزاد اسلامی واحد تبریز انتخاب شدند و پرسش‌نامه‌های 6 عاملی شخصیت هگزاکو (Hexaco) و پرسش‌نامه حمایت اجتماعی ادراک شده (Multidimensional scale of perceived social support) را تكميل کردند. تحلیل داده‌ها به کمک نرم‌افزارهای SPSS16 و LISREL8.54 و به‌روش آزمون تحلیل واریانس چندراهه (MANOVA) و تحلیل مسیر انجام شد. نتايج: نتایج پژوهش نشان داد که بین دو گروه، از نظر ویژگی‌های شخصیتی و حمایت اجتماعی تفاوت معناداری وجود دارد (05/0P<) و حمایت اجتماعی نقش تعديل‌كننده در بروز نشانگان بالینی بعد از شکست عاطفی دارد. نتيجه‌گيري: ویژگی‌های شخصیتی و حمایت اجتماعی در روابط عاطفی هر فرد دخیل مي‌باشد و نحوه برخورد با چالش‌های پیش‌رو در این نوع روابط را پیش‌بینی می‌کنند. بعد از شکست عاطفی، اگر خانواده بتواند فرزندان را با حمایت مستقیم و غیرمستقیم از این بحران خارج کند، فرد این داغدیدگی را به‌طور طبیعی می‌گذراند و نشانگان بالینی کمتر تداوم مي‌یابد.

Title: ساخت و طراحی سامانه دارویی آهسته رهش آسیکلوویر در مقیاس نانو

http://knh.shmu.ac.ir/index.php/site/article/view/59

مقدمه: تحقیق حاضر به‌منظور توسعه سامانه‌هاي نوین آزادسازي دارو بر پایه نانو ذرات صورت گرفته است. مواد و روش‌ها: در این تحقیق با استفاده از پلیمر طبیعی کیتوزان، که به‌علت خاصیت زیست سازگاري و زیست تخریب‌پذیري بالا یکی از مناسب‌ترین مواد براي استفاده در زمینه‌هاي دارورسانی می‌باشد، نسبت‌به تهیه ذرات در مقیاس نانومتر با استفاده از روش ژل شدن یونی اقدام شد. پارامترهای مطالعه شده در تعیین اندازه ذرات با استفاده از روش آماری دی اپتیمال، شامل غلظت دارو، نسبت غلظت کیتوزان به تری‌پلی‌فسفات و pH محلول دارو- پلیمر می‌باشند. جهت تعیین اندازه نانو ذرات حاصله از دستگاه پراکنش نور دینامیک، جهت بررسی شکل نانوذرات از میکروسکوپ الکترونی نگاره، برای بررسی ساختار تشکیل‌دهنده نانوذرات از طیف سنجی مادون قرمز و برای تعیین مقاومت حرارتی از آنالیز وزن‌سنجی حرارتی استفاده شد. نتايج: نانوذره بهینه با مشخصات اندازه ذره 24/3±132 نانومتر، پتانسیل زتای 2/87±32 میکرو ولت، شاخص توزیع اندازه ذرات برابر با 05/0±159/0 و میزان داروی بارگذاری شده 35/4±85 درصد به‌دست آمد. همچنین مطالعه رهایش آسیکلوویر در محیط آزمایشگاه میزان 17/81% در طول 48 ساعت را نتیجه داد. نتیجه‌گیری: نانوذرات آسیکلوویر تهیه شده در شرایط بهینه با اندازه کوچک و ساختار کروی شکل و همچنین پایداری حرارتی بالا، می‌توانند به‌عنوان حاملی مناسب برای مصارف دارو رسانی به‌کار روند.

Title: ارزیابی کارایی سیستم تهویه مکنده موضعی در کنترل گردوغبار Fe2O3 منتشره در هوای محیطی واحد سرند اکساید صنعت فولاد

http://knh.shmu.ac.ir/index.php/site/article/view/451

مقدمه: تا كنون راهکارهاي مختلفی براي کاهش تماس کارگران با آلاینده‌های شیمیایی و يا کنترل انتشار آنها، ارائه شده‌است. سیستم تهویه مکنده موضعی متداول‌ترين نوع کنترل‌های مهندسی است که بر دیگر روش‌های کنترلی ترجیح داده می‌شود. این مطالعه با هدف تعیین کارایی سیستم تهویه مکنده موضعی در کنترل ذرات منتشره در هوای محیطی واحد سرند اکساید در صنعت فولاد انجام شد. مواد و روش‌ها: در اين مطالعه توصیفی، به‌منظور ارزیابی عملکرد سیستم تهویه موضعی واحد موردنظر به چهار قسمت اصلی: طبقه همکف، طبقه سرندها، طبقه هود 1 و پلتفرم هودهاي 15، 16 و 17 تقسیم گردید. 36 نمونه هوا به‌روش 600NIOSH – (سیکلون) جهت نمونه‌برداری و اندازه‌گیری از گردوغبارهای قابل تنفس و گردوغبار کلی در وضعیت‌هاي روشن و خاموش سيستم تهويه جمع‌آوري گردید. نتایج: نتایج نشان داد که طبقه هود 1 دارای بیشترین غلظت گرد‌و‌غبار کلی به‌ترتیب با میانگین و دامنه تغییرات  mg/m3(47/434-1/118) 3/271 (تقریباً 2/18 برابر میزان استاندارد) و راندمان کنترل 9/3% بوده است. طبقه سرندها کمترین غلظت را به‌ترتیب با میانگین و دامنه تغییرات mg/m3 (51/31-95/8) 77/20 (تقریباً 4/1 برابر میزان استاندارد) و راندمان 35/29% در وضعیت سیستم روشن دارا بود. میانگین غلظت در کل واحد  mg/m3 (63/234-77/20) 6/127 و راندمان کلی سیستم در حدود 96/7% بود (mg/m3) 15mg/m3,PEL-OSHA: 10 (TLV-ACGIH:). نتیجه‌گیری: راندمان سیستم در قسمت‌های مختلف متفاوت بوده و در کل سیستم تهویه موضعی مورد مطالعه از كارآيي لازم برخوردار نبود که آن را می‌توان به فقدان برنامه منظم و زمان‌بندی شده نگهداری و پايش سیستم و همچنین تغییرات نرخ تولید نسبت داد.

Title: گزارش يك مورد پلاستيك دفورميتي استخوان فيبولا به همراه شكستگي تيبياي همان طرف در فرد بالغ

http://knh.shmu.ac.ir/index.php/site/article/view/489

پلاستیک دفورمیتی به تغییر شکل ثابت استخوان به‌دنبال اعمال نیرو گفته می‌شود که این پدیده در بزرگسالان نادر می‌باشد و در بچه‌ها شایع‌تر است. در این گزارش یک مورد پلاستیک دفورمیتی فیبولا همراه با شکستگی تیبیای همان طرف را در فردی بالغ معرفی می‌کنیم که به‌دنبال اصابت نیرویی مستقیم رخ داده است. در معاینه اولیه علایم حیاتی پایدار بود و در گرافی‌های انجام شده شکستگی ساق به‌همراه تغییرشکل پلاستیک فیبولا، مشهود بود. جهت درمان جااندازی بسته فیبولا و سپس فیکساسیون داخلی تیبیا انجام شد.
